# Defibrinogen Therapy for Acute Ischemic Stroke: 1332 Consecutive Cases

**DOI:** 10.1038/s41598-018-27856-6

**Published:** 2018-06-22

**Authors:** Jing Chen, Dalong Sun, Mingli Liu, Shufan Zhang, Chuancheng Ren

**Affiliations:** 1 Departments of Neurology, Shanghai Fifth People’s Hospital Affiliated to Fudan University, Minhang District, Shanghai, China; 2 Division of Gastroenterology, Department of Internal Medicine, Zhongshan Hospital Affiliated to Fudan University, Xuhui District, Shanghai, China; 3 Departments of Neurology, Shanghai East Hospital Affiliated to Tongji University, Pudong New Area, Shanghai, China

## Abstract

This study aimed to examine the effectiveness of defibrinogen therapy on functional recovery and safety among 1332 consecutive ischemic stroke patients who had not received intravenous thrombolysis with recombinant tissue plasminogen activator. Stroke patients undergoing conservative and relatively individualized multiple-day dosing regimens of defibrinogen therapy between January 1, 2008 and May 30, 2016 were enrolled. Data were analyzed according to functional success (Barthel Index of 95 or 100, mRS of 0 or 1) and safety variables (intracranial hemorrhage, mortality and stroke recurrence). At 12 months, 18.62% (203/1087) of patients were lost to follow-up. The functional success rates were 39.84% (526/1320) and 42.23% (459/1087) as assessed by BI at 3 months and 12 months, respectively. Fifteen patients had asymptomatic intracranial hemorrhage within 24 hours after the initial defibrase administration. During the 14 days after hospitalization, 12 patients were diagnosed with symptomatic intracranial hemorrhage (sICH) and a total of 12 patients died from all causes. At 3 months, 56 patients were dead and 21 patients had recurrent stroke. The percentage of death and recurrence of stroke at 12 months were 6.81% and 3.22%, respectively. Results from the historical control showed no significant differences of functional success were detected between the patients treated with rt-PA within 6 hours of stroke onset in NINDS II and the patients treated with defibrase within 6 hours after stroke in the present study. The multiple-day dosing regimen of defibrinogen therapy using defibrase applied in the present study could achieve functional improvement among acute ischemic stroke patients, with low risks of mortality when compared with other similar studies. However, the efficacy and safety of such a defibrinogenating therapy is needed to be verified by RCTs with large sample size.

## Introduction

Stroke is a major cause of death and adult disability throughout the world^1–3^. Currently, at organized stroke units, intravenous thrombolysis with recombinant tissue plasminogen activator (IV rt-PA) administered within 3 to 4.5 hours after symptom onset and endovascular therapy with or without intravenous thrombolysis within 6 hours are proven to be beneficial in acute ischemic stroke^4–7^. However, the narrow therapeutic window and low recanalization rate in proximal vessel occlusions of intravenous thrombolysis and the limitation of application to acute stroke patients with posterior circulation occlusion restrict the utility of these specific interventions^8–10^. Thus, new effective therapeutic strategies must be explored.

Epidemiological studies have established that elevated fibrinogen levels are independently and highly associated with stroke and peripheral arterial disease^11–13^. Studies found strong correlations between high initial fibrinogen levels in acute ischemic stroke patients and poor prognosis for functional outcomes^14,15^. Fibrinogen-depleting agents lower blood plasma fibrinogen levels, reduce blood viscosity, increase local blood flow and hence inhibit further thrombus formation or extension^16–18^. This drug-induced hypofibrinogenemia also promotes release of plasminogen activator with concomitant reductions in plasmin inhibitor and plasminogen activator inhibitor^19^. Fibrinogen-depleting agents have been used as reperfusion therapy for treatment of deep vein thrombosis, central retina venous thrombosis and peripheral vascular disorders since the 1970s in Europe and Canada^20–22^. However, prior randomized controlled trials (RCTs) reported inconsistent conclusions in terms of influence of fibrinogen depleting agents on functional outcomes of acute stroke patients^18,19,23–29^. Some studies showed fibrinogen depleting agents delivered in acute ischemic stroke patients did not improve them outcome and revealed a tread to increase bleeding^23,24,29^, while other researchers demonstrated fibrinogen depleting agents had a favorable benefit-risk profile for patients with acute ischemic stroke^18,19,25–28^. In the latest meta-analysis of fibrinogen depleting agents for acute ischemic stroke^30^, results showed that fibrinogen depleting agents marginally reduced the proportion of patients who were deed or disabled at the end of follow-up (risk ratio (RR): 0.95; 95% confidence interval (CI): 0.90–0.99; *P* = 0.02) and decreased the stroke recurrences (RR: 0.67; 95% CI 0.49–0.92; *P* = 0.01)^30^. In the meta-analysis, although the risk of symptomatic intracranial hemorrhage (sICH) was higher in fibrinogen depleting agent treatment group compared with the control group^30^, the risk could reduce as long as fibrinogen levels and other coagulation values were carefully controlled^18^. Moreover, factors such as time to treatment, dosing regimen and patient selection may influence the results^16,30^.

Hence, in the present study, which aimed to investigate the effectiveness and safety of a conservative and relatively individualized multiple-day dosing regimen of defibrinogenating agents in patients with ischemic stroke within 72 hours of recognized symptom onset, plasma fibrinogen level and global coagulation tests such as activated partial thromboplastin time (APTT), prothrombin time (PT), thrombin time (TT) were monitored carefully during each use of defibrase to reduce bleeding events, such as sICH and to confirm the safety. What’s more, we applied defibrase as interventional fibrinogen depleting agent other than ancrod (another kind of fibrinogen depleting agent) to reduce the risk of sICH, which in accord with the subgroup analysis of meta-analysis showed defibrse tend to be associated with the lower risk of sICH (RR: 1,15; 95% CI: 0.60–2.20) than ancrod (RR: 3.56; 95% CI: 2.15–5.87)^30^.

## Methods

### Study participants

This study was performed in compliance with the Declaration of Helsinki and approved by the Independence Ethics Committee of the Shanghai Fifth People’s Hospital Affiliated to Fudan University (ethic approval number: 2017-ETRE-059).

We conducted a retrospective review of a prospectively obtained dataset on all acute ischemic patients treated conservatively in our hospital from January 1, 2008 to May 30, 2016 who received defibrase (a defibrinogenating agent extracted from Agkistrodon acutus venom, 5 U/vial). Adults with an acute moderate or severe neurological deficit (pretreatment NIHSS 5–22) suggestive of ischemic origin were eligible for defibrinogen therapy. Defibrinogen therapy could be given within 72 hours after stroke symptom onset. Subjects with clinical or neuroimaging evidence of intracranial extravascular blood or obvious bleeding tendencies, or with pretreatment plasma fibrinogen levels below 1.5 g/L or persistent hypertension (systolic blood pressure >180 mmHg or diastolic blood pressure >110 mmHg), were excluded (Appendix 1). Patients had used or intended to use of thrombolytic agents including IV rt-PA were excluded. The patients who were not qualified to receive IV rt-PA, such as patients were missed the therapeutic window of IV rt-PA, patients or family members were unwilling to receive rt-PA and patients were over 80 years of age, were considered receiving the defibrinogen therapy.

Written informed consent documents were signed by the patients or their representatives before treatment.

### Dosing regimen of defibrase

**Figure 1:**
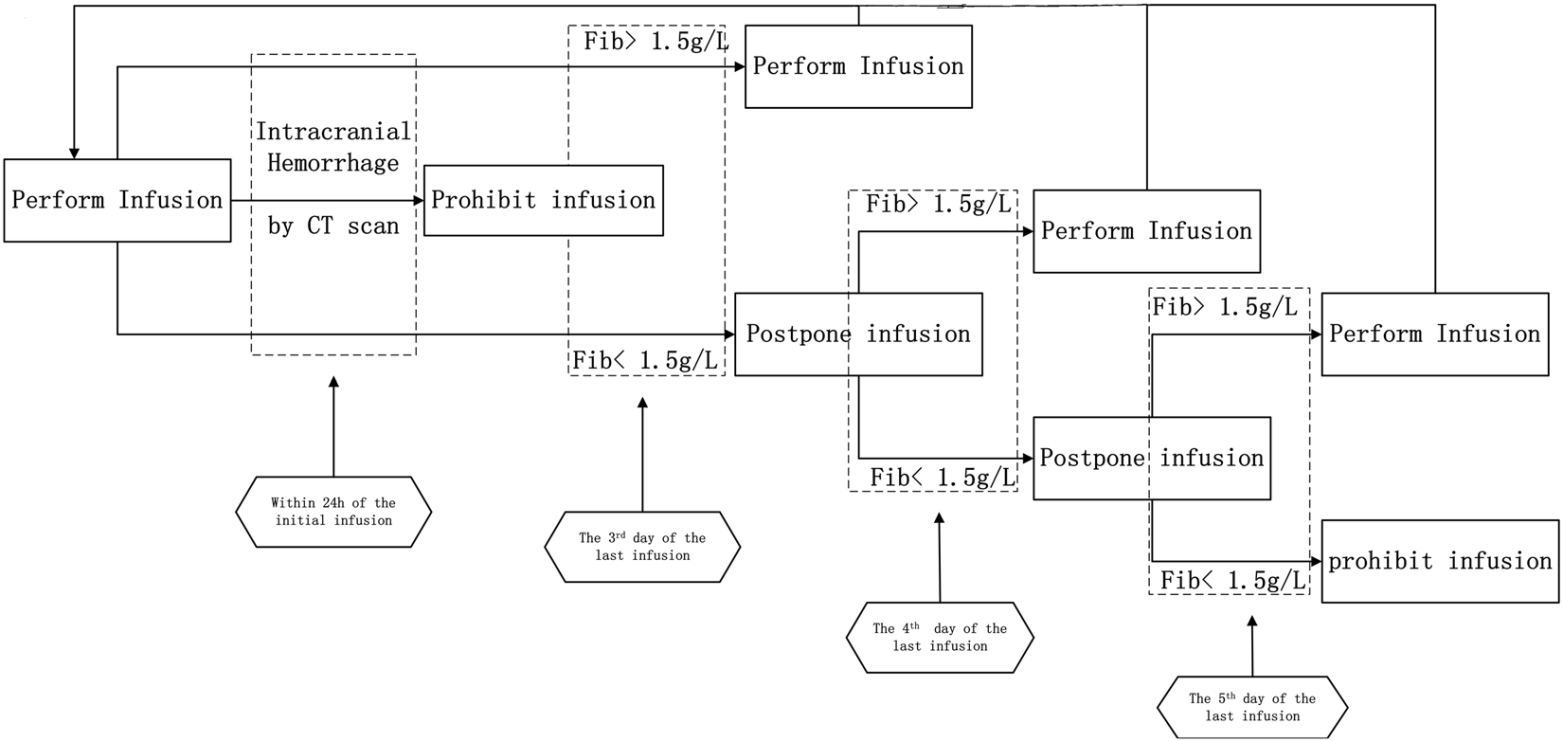
A flowchart of the dosing regimen.

A conservative and relatively individualized multiple-day dosing regimen of defibrase was adopted. Theoretically, each eligible patient should receive intravenous infusions of defibrase three times, on the 1^st^, 3^rd^ and 5^th^ days (defining the day of the initial infusion as the first day). However, the number of times and time interval of infusion depended on the concrete specifics of the situation. Brain CT scanning was conducted within 24 hours after initial infusion of defibrase. If the results of the CT scan presented intracranial hemorrhage, the subsequent infusions were prohibited. If not, the second or third infusion was given based on the fibrinogen level measured on the 3^rd^ day after the previous infusion. On the one hand, theoretically, each eligible patient should receive intravenous infusions of defibrase three times at the 1st, 3rd and 5th day (presumed that the day receiving the initial infusion as the first day). But the number of times and time interval of infusion depended on fibrinogen level. Fibrinogen level was measured before every defibrase infusion, when fibrinogen level was less 1.5 g/L, defibrase infusion was prohibited absolutely (lower than 1.5 g/L for three consecutive days, measured once a day) or delay until next measurement of fibrinogen level was more than 1.5 g/L (lower than 1.5 g/L for once or twice, measured once a day). Hence, patients may receive the defibrase infusion once, twice or three times, and patients received the defibrase infusion in the first day for the initial infusion, the 3th, or 4th or 5th day for the second infusion (Fig. 1). On the anther hand, when fibrinogen level was between 1.5 g/L and 2.0 g/L, the dose was 5 U; when fibrinogen level was over 2.0 g/L, the dose was 10 U (Fig. 1). Defibrase (10 U or 5 U) was diluted into 250 mL saline and administered at the same infusion rate (30 drops per min). Eligible patients were not allowed to receive thrombolytic agents, antiplatelet drugs, anticoagulants, heparin, or other agents that might influence the fibrinolytic system. After completion of defibrinogen therapy, patients were permitted to receive prophylactic measures (such as antiplatelet or anticoagulant drugs) as required.

### Data collection and outcome measures

All pretreatment and posttreatment data were collected from the hospital’s electronic medical system and recorded in a review-board-approved electronic database. The data parameters collected from pretreatment included age, gender, smoking status, blood pressure, time to treatment, National Institute of Health Stroke Scale (NIHSS) score, and history of hypertension, diabetes mellitus, dyslipidemia, atrial fibrillation, transient ischemic attack (TIA) and stroke. Hematology parameters and global coagulation tests such as APTT, PT, and TT were measured before treatment and on the 14^th^ day after admission. Plasma fibrinogen level was measured before pretreatment (Fib-i), on the 3^rd^ day after each infusion of defibrase (Fib-1st, Fib-2nd, Fib-3rd) and on the 14^th^ day after admission (Fib-14d).

The efficacy end points of efficacy included the Barthel Index (BI) and the modified Rankin scale (mRS) at 3 months and 12 months after defibrinogen therapy. A BI score of 95 or 100 and an mRS score of 0 or 1 at 3 months and 12 months were defined as functional success. The safety end points consisted of asymptomatic intracranial hemorrhage within 24 hours after the initial defibrase treatment (detected by a repeat CT scan); symptomatic intracranial hemorrhage (sICH); bleeding at other sites; coagulation tests and special hematology profiles during the first 14 days after admission; mortality at the 14^th^ days after admission, 3 months and 12 months; and recurrence of stroke at 3 months and 12 months.

### Statistical analyses

The normality of the data distribution was evaluated by the Shapiro-Wilk test. Data were presented as the means, standard deviation, medians or ranges for continuous variables and percentages for categorical variables. Descriptive statistical analyses and paired-sample *t*-tests were performed for demographic data, hematology and coagulation function indicators. Distribution of the percentages of functional success in difference variables were analyzed by using the chi-squared statistic, Fisher’s exact test or the Cochran-Mantel-Haenszel chi-squared test. Collinearity diagnosis tests were performed to determine whether there was multicollinearity among variables (age, initial NIHSS score, smoking or not, initial fibrinogen level, time to treatment, number of times defibrase was given and dose of defibrase), which may influence the independent roles of variables or the degree of correlation between them in the multivariate regression model. Logistic regression analysis was used to estimate odds ratios for functional success at 12 months. Potential covariates, such as age, gender, smoking, high risk of factors, history of prior TIA or stroke, initial NIHSS score, initial fibrinogen level, time to treatment, the number of uses of defibrase and the total dose of defibrase, were first calculated in univariate analyses, and factors that showed significant in univariate analyses were furtherly introduced to the multivariable modeling. The statistical tests were 2-sided and at the 5% significance level. Analyses were conducted using IBM SPSS version 19.0 statistical software.

## Results

### Demographics and dosing characteristics

A total of 1332 patients who received defibrinogen therapy from January 1, 2008 to May 30, 2016 were enrolled in this study. The patients had an average age of 65.4 years, and 55.3% were men. The median pretreatment NIHSS score was 9 with a range of 6 to 22. The median time to treatment was 37 hours, with only 7.0% of patients receiving defibrase within 6 hours of symptom onset. The mean level of initial fibrinogen was 3.2 g/L, and the majority of patients had levels of over 2.0 g/L (91.4%). Nine hundred twenty-twp (69.1%) of the patients received defibrase three times. The most common dose of defibrase (32.9% of patients) was 20 U. The complete demographics and dosing characteristics are listed in Table 1.

**Table 1:** Demographics and dosing characteristics.

	Total
No.	1332
Age, y, mean (SD)	65.4 (10.8)
≦60, n (%)	423 (31.8)
61–79, n (%)	766 (57.5)
≧80, n (%)	143 (10.7)
Male, n (%)	737 (55.3)
Smoking, n (%)	434 (32.6)
Hypertension, n (%)	719 (54)
Diabetes mellitus, n (%)	496 (37.2)
Atrial fibrillation, n (%)	350 (26.3)
Dyslipidemia, n (%)	389 (29.2)
History of prior TIA, n (%)	231 (17.3)
History of prior stroke, n (%)	197 (14.8)
Blood pressure, mmHg, mean (SD)	157.65 (12.5)/88.42 (8.2)
Pretreatment NIHSS, median [range]	9 [6–22]
5–7, n (%)	396 (29.7)
8–15, n (%)	695 (52.2)
≧16, n (%)	241 (18.1)
Time to treatment, h, median [range]	37 [4–72]
≦6, n (%)	93 (7.0)
7–12, n (%)	162 (12.2)
12–24, n (%)	221 (16.5)
25–48, n (%)	457 (34.3)
49–72, n (%)	399 (30.0)
Initial fibrinogen level, g/L, mean (SD)	3.2 (1.1)
1.5–2.0, n (%)	115 (8.6)
≧2.0, n (%)	1217 (91.4)
Number of defibrase infusions	
1, n (%)	197 (14.8)
2, n (%)	213 (15.9)
3, n (%)	922 (69.3)
Dose of defibrase	
5 U, n (%)	93 (7.0)
10 U, n (%)	136 (10.2)
15 U, n (%)	211 (15.8)
20 U, n (%)	438 (32.9)
25 U, n (%)	350 (26.3)
30 U, n (%)	104 (7.8)

Abbreviations: SD, standard deviation; TIA = transient ischemic attack; NIHSS, National Institute of Health Stroke Scale.

### Hematology and coagulation function

Compared with pretreatment, the mean value of plasma viscosity (1.7 to 1.3 mPa/s), PLT (224 to 210 × 10^9^/L), hemoglobin (141 to 138 g/L), Hct value (46.4 to 42.4%), platelet aggregation rate (65.9 to 57%) were all significantly lower on the 14^th^ day after treatment. The changes in the mean values of APTT, PT and TT between pretreatment and the 14^th^ day after admission attained statistical significance (difference in mean score: 8.60 s, 0.74 s and 3.35 s, respectively). All the hematologic and coagulation function parameters measured in the pretreatment and at the 14^th^ day after admission were within the normal range according to our hospital’s test standard (Appendix 2).

**Figure 2:**
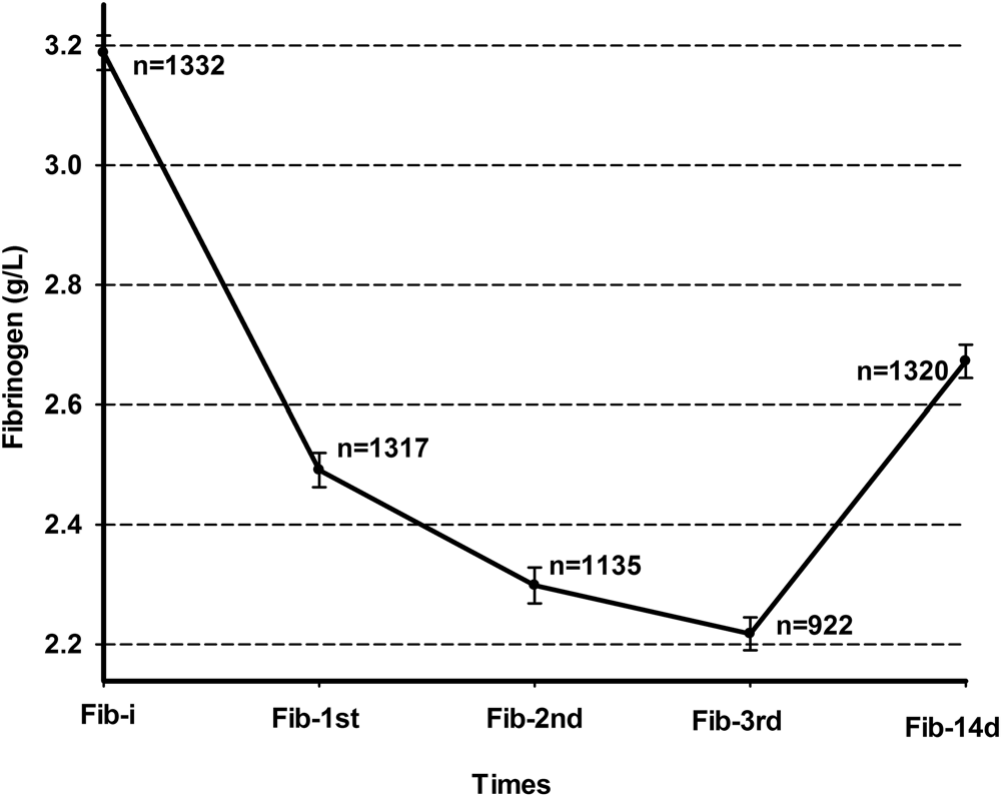
Changes in plasma fibrinogen levels. Fib-i: fibrinogen level measured at pretreatment; Fib-1st: fibrinogen level measured on the 3^rd^ day after the first infusion of defibrase; Fib-2nd: fibrinogen level measured on the 3^rd^ day after the second infusion of defibrase; Fib-3rd: fibrinogen level measured on the 3^rd^ day after the third infusion of defibrase; Fib-14d: fibrinogen level measured on the 14^th^ day after admission.

Plasma fibrinogen levels consistently reduced from 3.18 ± 1.05 to 2.49 ± 1.03 to 2.29 ± 1.01 g/L from pretreatment (n = 1332) to the 3^rd^ days after the first (n = 1317) and second infusions (n = 1135) of defibrase, reaching the lowest level after the third infusion (2.21 ± 0.83 g/L, n = 922). However, the plasma fibrinogen level increased to 2.67 ± 1.01 g/L on the 14^th^ day after admission (n = 1320) (Fig. 2).

### Outcomes and follow-up

**Figure 3:**
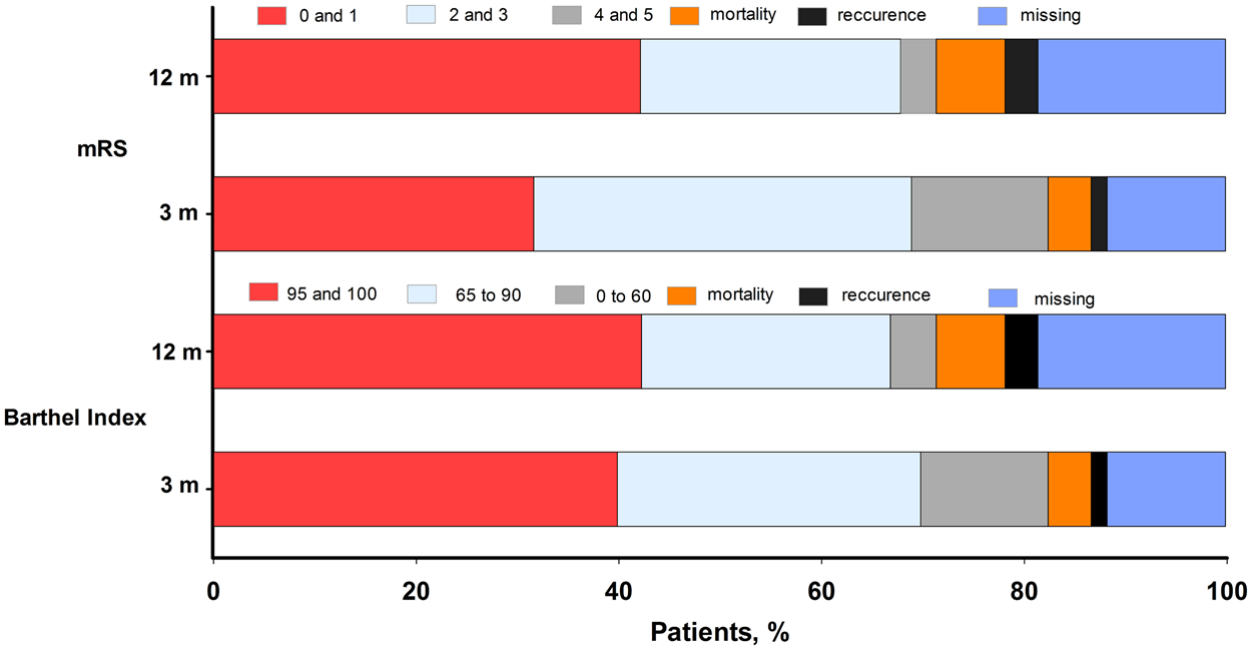
Overall distributions of Barthel Index score and mRS at 3 months and 12 months. mRS score of 0 and 1, and Barthel Index score of 95 and 100 were defined as functional success. For mRS, grades 0 or 1, 2 or 3, and 4 or 5 are considered as no or minimal disability, moderate disability, and severe disability, respectively. For Barthel Index, the higher scores demonstrated less severity of stroke. mRS: modified Rankin scale.

Among the 1332 patients, within 24 hours of initial infusion of defibrase, repeated CT scan discovered that 15 patients had asymptomatic intracranial hemorrhage. During the 14 days after hospitalization, 12 were patients diagnosed with sICH, 12 patients died (five died of sepsis, three of sICH, three of severe pneumonia, and one from a heart attack), and 16 patients had bleeding at other sites (seven for ecchymosis or petechiae or bleeding gums, four for gastrointestinal bleeding, three for epistaxis, an two for hematuria). One hundred fifty-six patients (11.82%) were lost to follow-up after 3 months, and more patients (18.68%) failed to follow-up after 12 months. The differences of demographic (age, gender) and baseline clinical characteristics (high risk factors, stroke associated past history, hematology and coagulation function parameters, fibrinogen level and neurological function and condition of defibrase therapy) between the patients who were failed to follow-up and patients who were followed up at 3 months and 12 months were calculated. No significant differences were found between the two groups as listed in Appendix 3. The functional success rates were 39.84% assessed by BI score of 95 or 100 and 31.59% assessed by an mRS of 0 or 1 among 1320 patients at 3 months. At 12 months, the functional success rates were higher, reaching 42.23% by BI score and 42.13% by mRS among 1087 patients. At 3 months, among 1320 patients, 56 patients had died and 21 patients had suffered another stroke, accounting for 4.24% and 1.59%, respectively. The death and recurrence rate of stroke were 6.81% and 3.22% respectively, in 1087 patients at 12 months. The overall distribution of efficacy and safety outcomes, including mortality, recurrence rate of stroke and follow-up information at 3 months and 12 months, is shown in Fig. 3.

Appendix 4 shows the distribution by percentage of functional success of different initial fibrinogen levels, the numbers of administrations of defibrase, total doses of defibrase and time-to-treatment categories at 3 months and 12 months. Significant difference was found in different initial fibrinogen level (*P* = 0.017) and number of times of administration of defibrase (*P* = 0.049) measured by mRS of 0 or 1 at 3 months. The rates of functional success measured both by a Barthel Index of 95 or 100 and by mRS of 0 or 1 in different total dose of defibrase at 3 months and 12 months were significantly different (*P* < 0.01).

There was no obvious multicollinearity among variables, including age, initial NIHSS score, smoking or not, initial fibrinogen level, time to treatment, number of defibrase administrations and dose of defibrase. Factors of age, smoking, NIHSS score, initial fibrinogen level, time to treatment, the number of uses of defibrase and the total dose of defibrase showed significant in univariate analyses, and such factors were furtherly introduced to the multivariable analysis. Table 2 showed the results of multivariable analyses of the relation between demographics and dosing characteristics and the rate of functional success at 12 months. For the Barthel Index, analyses of the association between NIHSS score, initial fibrinogen (1.5~2.0 g/L), 10 U of defibrase, 15 U of defibrase and functional success yielded an adjusted odds ratio of 1.269 (95% CI: 1.022 to 1.576; *P* = 0.031), 1.697 (95% CI: 1.028 to 2.800; *P* = 0.039), 0.452 (95% CI: 0.210 to 0.9720; *P* = 0.042) and 0.520 (95% CI: 0.282 to 0.959; *P* = 0.036), respectively. For mRS, analyses of the association between smoking, 5 U, 15 U, 20 U, 25 U dose of defibrase showed an adjusted odds ratio of 1.760 (95% CI: 1.261 to 2.456; *P* = 0.001), 0.302 (95% CI: 0.120 to 0.761; *P* = 0.011), 0.225 (95% CI: 0.118 to 0.427; *P* < 0.001), 0.311 (95% CI: 0.177 to 0.545; *P* < 0.001) and 0.467 (95% CI: 0.267 to 0.815; *P* = 0.007), respectively.

**Table 2:** Association of demographics and dosing characteristics with functional success at 12 months.

Variable	Functional Success (Barthel Index of 95 or 100 at 12 months)				Functional Success (mRS of 0 or 1 at 12 months)			
	775 Subjects, 459 Events				775 Subjects, 458 Events			
	OR	Lower CI	Upper CI	*P* Value	OR	Lower CI	Upper CI	*P* Value
Age/y	0.976	0.77	1.237	0.839	0.887	0.695	1.131	0.333
NIHSS Score	0.269	0.022	0.576	0.031	1.03	0.824	1.286	0.797
Smoking (No)	1.222	0.887	1.682	0.221	1.76	1.261	2.456	0.001
Initial Fibrinogen (1.5–2.0 g/L)	1.697	1.028	2.8	0.039	1.524	0.915	2.539	0.106
Time to Treatment								
49–72 h	Ref	…	…	0.478	Ref	…	…	0.267
0–6 h	1.189	0.6	2.356	0.62	0.746	0.376	1.481	0.403
7–12 h	1.363	0.722	2.574	0.339	0.596	0.315	1.128	0.112
13–24 h	1.655	0.868	3.154	0.126	0.701	0.367	1.339	0.282
25–48 h	1.423	0.703	2.881	0.327	0.486	0.237	0.995	0.058
Number of defibrase uses								
3	Ref	…	…	0.255	Ref	…	…	0.916
1	1.602	0.855	3	0.141	1.128	0.602	2.116	0.707
2	1.3	0.826	2.046	0.256	1.071	0.669	1.714	0.775
Dose of defibrase								
30 U	Ref	…	…	0.193	Ref	…	…	0
5 U	0.53	0.215	1.308	0.168	0.302	0.12	0.761	0.011
10 U	0.452	0.21	0.972	0.042	0.526	0.244	1.134	0.101
15 U	0.52	0.282	0.959	0.036	0.225	0.118	0.427	0
20 U	0.658	0.383	1.129	0.128	0.311	0.177	0.545	0
25 U	0.833	0.487	1.426	0.505	0.467	0.267	0.815	0.007

Abbreviations: OR = odd ratio; CI = confidence interval; NIHSS, National Institute of Health Stroke Scale.

### Differences in functional success between the present study and the NINDS II

A landmark trial published in 1995 by the *National Institute of Neurological Diseases and Stroke (NINDS)* about rt-PA for treating acute ischemic stroke within 6 hours from stroke onset demonstrated that the likelihood of functional improvement (BI score of 95 or 100; mRS of 0 or 1) at three months was higher in the rt-PA treatment group than the control group, even though the difference of early stage outcome measured by NIHSS at 24 h after the onset of stroke was not significant between the two groups^31^. In NINDS II, there were 168 patients treated with rt-PA within 6 hours after stroke onset, and 84 of them obtained BI of 95 or 100, and 66 of them achieved mRS of 0 or 1. We compared the results that gained functional success at 3 months between the patients treated with defibrase within 72 hours after stroke onset in our study and 168 patients treated with rt-PA within 6 hours in NINDS II. As showed in Table 3, for outcome of BI of 95 or 100, NINDS II shows more effect in functional improvement than our study (*P* = 0.030); for outcome of mRS of 0 or 1, the difference between our study and NINDS II was not significant (*P* = 0.067). Furthermore, we compared the results of functional success from the 93 patients treated within 6 h after stroke onset in our study with that from 168 patients in NINDS II. No significant differences between our study and NINDS II were found (for BI: *P* = 0.194; for mRS: *P* = 0.423; Table 3). Lastly, the differences of mortality rate at 3 months between patients treated within 72 hours in our present study and patients treated within 6 hours in the study of NINDS, including NINDS I (n = 144) and NINDS II (n = 168) were calculated. Results showed that the rate of mortality was lower in our study (56/1320) than that in NINDS (54/312) at 3 months (*P* < 0.001).

**Table 3:** Comparisons of functional success between our present study and NINDS II at 3 months.

	TTT ≦ 6 h		*P*	TTT ≦ 72 h		*P*
	Present study NINDS II			Present study NINDS II		
	93	168		1320	168	
BI of 95 or 100, n (%)	37 (39.78)	84 (50.00)	0.194	526 (39.84)	84 (50.00)	0.030
mRS of 0 or 1, n (%)	31 (33.33)	66 (39.00)	0.423	417 (31.59)	66 (39.00)	0.067

Abbreviations: TTT = time to treatment; NINDS = National Institute of Neurological Diseases and Stroke; BI = Barthel Index; mRS = modified Rankin score.

## Discussion

A homogeneous series of 1332 consecutive patients receiving a conservative and relatively individualized multiple-day dosing regimen of defibrase were observed in term of efficacy and safety in this study. The patients in this series were chosen with caution. The multiple-day dosing regimen extended the therapy window up to 72 hours after symptom onset. Defibrase has been used to treat strokes for decades. In previous studies, ischemic stroke patients were administered defibrinogenating agents (ancrod or defibrase) within 3 to 48 hours from stoke onset^18,23–28^. Additionally, a large multicenter randomized controlled trial was conducted to explore the effectiveness and safety of defibrase delivered within 24 hours after the onset of stroke symptom^29^. Fibrinogen level was regarded as a vital monitoring indicator in the current study. Whether to use of defibrase to treat stroke patients relied on plasma fibrinogen level measured before delivery. The variability of patients treated at different time points, with different doses, and with different courses of treatment were all depending on the plasma fibrinogen level. The reason for designing such a seemingly individualized protocol is to make sure each defibrase infusion is safety and cause less bleeding events. Strict patient selection, extended therapy window and cautious dosing regimen were applied to allow more eligible patients to receive defibrinogen therapy and to reduce the risk of hemorrhage as far as possible. This case series reported a conservative, moderate and relatively individualized defibrinogen therapy for the treatment of ischemic stroke patients in a time window of 72 hours from stroke onset for the first time.

In terms of Barthel Index score, according to worst-case-scenario intention-to-treat (ITT) analysis, the functional success rate at 3 months in our study was higher than that reported by the Cooperative Group for Reassessment of Defibrase in 2005 (39.84% versus 36.6%), in which patients also received defibrase as defibrinogenating agents within 12 hours of stroke onset^25^. However, other researchers have reported that functional success rate was higher than 39.84%, which included the patients whose Barthel Index score were less than 95 but at least equal to the pre-stroke value^27^. At 12 months, the functional success rate was further increased which was in line with the European Stroke Treatment with Ancrod Trial (ESTAT) report^24^. In addition, functional success was further investigated according to different categories. At 3 months, the differences of functional success rate assessed by mRS were statistically significant between groups with different initial fibrinogen level and among groups with different numbers of times treated with defibrase. Furthermore, significant differences were identified among groups with different total dose of defibrase for functional success rate both assessed by Barthel Index and mRS scores at 3 months and 12 months, however which doses was optimal for attaining functional success required further study. Unexpectedly, five different time-to-treatment groups had same effects on the functional success.

Regarding safety, the average of hematologic and coagulation function parameters at the 14^th^ day was lower than that at admission, but all at normal levels which may be due to blood dilution caused by liquid supplement. In addition, the reduction of plasma fibrinogen level might result in a decrease in plasma viscosity and platelet aggregation rate. Therefore, the decreases of ATPP, PT and TT might be the combined results of the above. During defibrinogen therapy, symptomatic intracranial hemorrhage and death from all causes were lower most of the reported results. It may largely result from the close monitoring of fibrinogen level during the process of defibrinogenating therapy. Mortality rate at 3 months and 12 months (4.24% at 3 months and 3.2% at 12 months) were also lower than that of a recent systematical review and meta-analysis of eight RCTs^16^, in which the death from all cause at the end of follow-up in the fibrinogen depleting agents group was as high as 15%. Moreover, the recurrence rate of stroke at 12 months was only 3.22%, which was lower than that reported by Liu *et al*. and Hao *et al*.^16,25^.

Results from the comparisons of our study and NINDS II displayed that patients treated with rt-PA within 6 hours after stroke onset were more likely to obtain functional success (BI of 95 or 100) than that treated with defibrase within 72 hours from stroke symptom onset at 3 months. But no significant differences of functional success were detected when compared the patients treated with rt-PA within 6 hours of stroke onset in NINDS II and the patients treated with defibrase within 6 hours after stroke in the present study. It may imply that time-to-treatment played key roles in treatment of acute ischemic stroke. It conformed to the opinion that the earlier the treatment, the more benefit it would achieve. Additionally, we may try to infer that the treatment of rt-PA used in the NINDS II and the therapy of multiple-day dosing regimen applied in the present study plays equal effects in improving neural function in treating acute ischemic stroke within 6 hours of onset if there were no significant differences of demographic and baseline clinical characteristics between the 93 patients in our current study and 168 patients in NINDS II. The result that the mortality rate at 3 month was lower in our present study than that in NINDS among patients treated within 6 hours after the onset of stroke may largely owe to the close monitoring of fibrinogen level during the process of defibrinogenating therapy.

Based on the findings, the current study seems to indicate that for those patients who are not qualified for acute intervention with IV rt-PA, defibrinogen therapy appears safe and has similar outcomes, especially when used early, to a group of patients administrated IV rt-PA for acute stroke, and the effectiveness and safety of this defibrinogen therapy for stroke patients in this case series significantly improved compared with previous defibrinogenating therapy studies. Although the reasons for this disparity are unclear, it might because of patient selection, time to treatment, and dosing regimen strategies.

Patient selection is the first variable to explore. All the cases in this series were consecutively collected over the course of eight years and five months and treated by the same medical team. Since defibrase was the only defibrinogenating agent provided in this case series, the selection bias should have been minimized. The age, gender and medical history of the patients represented in this series were similar to patients in other studies. Patients of non-smoking, lower NIHSS score at admission are more likely to acquire functional success. However, unlike other trials^25,29^, patients with atrial fibrillation or history of prior stroke were also included in this series. Therefore, patient selection may not play a critical role.

The second variable is the treatment window of within 72 hours after stroke onset. The well-known term of “time is brain” underlines the rapidity of loss of neural structures during an ischemic stroke. It was reported, on average, to be 13.6 billion synapses, 11.9 km of neuronal fibers and 1.9 billion of neurons with each passing minute^32^. Therefore, timely recanalization in the occluded artery often makes for the salvage of ischemic penumbra, the restoration of cerebral perfusion and improvement of clinical outcomes^33^. However, in the current study, the time window of 72 h was made to allow more eligible stroke patients to receive the defibrinogen therapy. The feasible bases on some studies, which reported that fibrinogen level of patients was consistently higher within 72 hours after stroke onset than that of healthy control and hyperfibrinogenemia predicted risk of death after ischemic stroke^34–36^. Although none of previous studies delivered defibrinogenating agents to acute ischemic stroke patients in such an extended time window, the subgroup analysis by time to treatment showed no statistically significant difference in functional success reported by this case series and other studies^24,27^. In addition, it was reported that if patients were treated earlier, they functional improvement was better^25^. Thus, it would be difficult to attribute the differences between the results from this series and those from other related studies to purely extended therapeutic time window factors.

Finally, the differences in dosing regimen should also be investigated. Defibrase has been proved to be effective in improving functional outcomes among Chinese patients with acute ischemic stroke by Liu *et al*.^25^. The differences in outcomes might arise from the differences in dosing regimen strategies of fibrinolytic drugs. In our study, the multiple-day dosing regimen of defibrase was applied and mainly aimed to reduce bleeding events, such as sICH. Results showed that the number of defibrase administrations appeared not to be associated with functional success. However, higher total dose of defibrase appears to be conducive to higher functional success rates (Appendix 4). We may indicate that whether the neural function can improve decides on total dose of defibrase other than the number of defibrase administrations, but the latter combined fibrinogen lever measured before each delivery could contribute to reassess and predict the risk of bleeding caused by defibrinogenating therapy and confirm the safety. Compared with the briefer but more intense dosing applied by David *et al*.^23^ and unified multiple-day dosing regimen adopted by most of other studies^24–27^, such multiple-day dosing regimen used in the current series were moderate and relatively individualized with the fundamental purpose not to lower plasma fibrinogen level to the desired level but to maintain a low normal level and its ultimate goal of increasing blood flow in the infarct site and reducing the risk of intracranial hemorrhage^37^.

Limitations were also applied in this series of cases studies. First, There were incomplete outcome data for 11.82% of patients at 3 months and 18.68% at 12 months because of loss to follow-up. Although analyses that compared the differences of demographic and baseline clinical charateristics between the patients failed to follow-up and patients followed up at 3 months and 12 months identified no significant differences. Relatively high loss to follow-up may intend to influence the evaluation of effectiveness and safety of multiple-day dosing regimen adopted in the current study. Second, because this study is limited due to the inherent weaknesses of a retrospective review, information such as stroke etiology and volume of stroke infarction lesion were not available for us. These potential confounders were not tried to be controlled by statistical analysis and may exert some influences on the results. Last but not least, the present study was a case series other than a controlled trial, and we should be more caution in interpreting the results because of the lack of control group. Although the historical control was performed, the effects of such comparison are far more less than that of concurrent controls due to variable factors, such as the different participant cohort and different study conditions.

## Conclusion

The multiple-day dosing regimen of defibrinogen therapy using defibrase applied in the present study could achieve functional improvement among acute ischemic stroke patients, with low risks of intracranial hemorrhage, mortality and stroke recurrence when compared with other similar studies. However, the efficacy and safety of such a defibrinogenating therapy is needed to be verified by RCTs with large sample size.

## Electronic supplementary material

Supplementary Information
