# Supplementary material for: Defibrinogen Therapy for Acute Ischemic Stroke: 1332 Consecutive Cases
Source: Sci Rep. 2018 Jun 22;8:9489. doi: 10.1038/s41598-018-27856-6 (PMC6014979; doi:10.1038/s41598-018-27856-6)
Supplement: Supplementary file 1 — Supplementary Information [file 41598_2018_27856_MOESM1_ESM.pdf]

# Supplementary Materials

Defibrinogen Therapy for Acute Ischemic Stroke: 1332 Consecutive Cases

Jing Chen[1*], Dalong Sun[2*], Mingli Liu[1*], Shufan Zhang[1], Chuancheng Ren[1,3]

[1]Departments of Neurology, Shanghai Fifth People's Hospital Affiliated to Fudan University, Minhang District, Shanghai, China

[2]Division of Gastroenterology, Department of Internal Medicine, Zhongshan Hospital Affiliated to Fudan University, Xuhui District, Shanghai, China

[3]Departments of Neurology, Shanghai East Hospital Affiliated to Tongji University, Pudong New Area, Shanghai, China

[*]Jing Chen, Dalong Sun and Mingli Liu contributed equally to the manuscript.

Correspondence

Chuancheng Ren, Departments of Neurology, Shanghai Fifth People's Hospital Affiliated to Fudan University, Minhang District, Shanghai, China; Departments of Neurology, Shanghai East Hospital Affiliated to Tongji University, Pudong New Area, Shanghai, 200235, China

Email: rccfns17@sina.com.

Inclusion Criteria

- Age ≧18 years
- Signs and symptoms of ischemic stroke (any vascular territory)
- Within 72 hours after recognized symptom onset
- 5 ≦ Pretreatment NIHSS ≦ 22
- Initial fibrinogen level ≧ 1.5g/L
- Written informed consent

Exclusion Criteria

- Coma
- Large area of infarction lesion or multiple ischemic lesions
- Clinical or neuroimaging evidence of intracranial hemorrhage
- Obvious bleeding tendency or abnormal of blood platelet count or coagulation function disorders
- History of severe brain trauma or intracranial hemorrhage or digestive tract hemorrhage
- Exposure to defibrinogenating or snake venom agents within 1 week
- Use or intended use of thrombolytic agent
- Persistent systolic blood pressure > 180 mmHg or diastolic blood pressure > 110 mmHg
- Associated severe primary disease of heart, liver, kidney or infectious diseases or hematological system or malignant tumor or severe progressive disease in any other system
- People with allergies or in pregnant

Appendix 1.Selection criteria for patients receiving defibrinogen therapy. Abbreviations: SD, standard deviation; NIHSS, National Institute of Health Stroke Scale

| | Pretreatment | At the 14th day | t |
|---|---|---|---|
| Hematology | | | |
| Plasma viscosity, mPa/s, mean (SD) | 1.7 (0.13) | 1.3 (0.12) | 67.62* |
| Hct value, % | 46.4 (0.09) | 42.2 (0.06) | 14.14* |
| Platelet aggregation rate, % | 65.9 (12.4) | 57 (12.4) | 17.89* |
| PLT, $\times 10^9$/L, mean (SD) | 224 (61.4) | 210 (60.3) | 5.76* |
| Hemoglobin, g/L, mean (SD) | 141 (21.9) | 138 (16.3) | 3.65* |
| Coagulation Function | | | |
| APTT, s, mean (SD) | 34.8 (5.1) | 26.3 (3.8) | 49.04* |
| PT, s, mean (SD) | 12.7 (3.9) | 11.9 (4.1) | 4.77* |
| TT, s, mean (SD) | 19.6 (2.4) | 16.3 (2.3) | 36.51* |

Appendix 2. Hematology and coagulation function parameters pretreatment and at the 14th day after admission. * The corresponding *P* value is less than 0.001 by paired samples t test. Abbreviations: SD=standard deviation, Hct=Hematocrit value, PLT=Platelet count, APTT=activated partial thromboplastin time, PT=prothrombin time, TT=thrombin time

| | 3 months | | | 12 months | | |
|---|---|---|---|---|---|---|
| | Loss (n= 156) | Not loss (n= 1164) | *P* | Loss (n= 203) | Not loss (n= 884) | *P* |
| Age, year | | | 0.783 | | | 0.309 |
| ≦ 60, n (%) | 50(32.1) | 368(31.6) | | 72(35.5) | 274(31.0) | |
| 61-79, n (%) | 87(55.8) | 674(57.9) | | 114(56.2) | 511(57.8) | |
| ≧ 80, n (%) | 19(12.2) | 122(10.5) | | 17(8.4) | 99(11.2) | |
| Male, n (%) | 85(54.5) | 645(55.4) | 0.827 | 106(52.2) | 483(54.6) | 0.532 |
| Smoking, n (%) | 44(28.2) | 384(33.0) | 0.231 | 63 (31.0) | 281(31.8) | 0.835 |
| Hypertension, n (%) | 81(51.9) | 643(55.2) | 0.612 | 103(50.7) | 487(55.1) | 0.262 |
| Diabetes mellitus, n (%) | 61(39.1) | 430(36.9) | 0.600 | 77(37.9) | 320(36.2) | 0.644 |
| Atrial fibrillation, n (%) | 36(23.1) | 308(26.5) | 0.366 | 56(27.6) | 229(25.9) | 0.623 |
| Dyslipidemia, n (%) | 46(29.5) | 339(29.1) | 0.925 | 56(27.6) | 259(29.3) | 0.628 |
| Plasma viscosity, mPa/s, mean (SD) | 1.71(0.14) | 1.70(0.12) | 0.477 | 1.69(0.12) | 1.70(0.13) | 0.760 |
| Hct value, % | 0.46(0.07) | 0.47(0.10) | 0.401 | 0.47(0.11) | 0.46(0.09) | 0.661 |
| Platelet aggregation rate, % | 67.45(13.04) | 65.65(12.38) | 0.091 | 64.64(13.23) | 65.89(12.02) | 0.192 |
| PLT, ×109/L, mean (SD) | 218(60.81) | 224(61.35) | 0.198 | 229.22(63.11) | 224.95(60.92) | 0.371 |
| Hemoglobin, g/L, mean (SD) | 142.27(24.78) | 141.33(21.42) | 0.613 | 141.55(15.19) | 141.25 (21.83) | 0.851 |
| APTT, s, mean (SD) | 35.04(4.78) | 34.83(5.17) | 0.634 | 35.28(4.70) | 34.75(5.27) | 0.198 |
| PT, s, mean (SD) | 12.76(2.62) | 12.73(4.02) | 0.913 | 12.72(1.82) | 12.78(4.63) | 0.839 |
| TT, s, mean (SD) | 20.03(2.51) | 19.60(2.39) | 0.630 | 19.65(2.31) | 19.62(2.39) | 0.860 |
| History of prior TIA, n (%) | 22(14.1) | 205(17.6) | 0.275 | 34(16.7) | 157(17.8) | 0.733 |
| History of prior stroke, n (%) | 23(14.7) | 171(14.7) | 0.986 | 22(10.8) | 134(15.2) | 0.113 |
| Blood pressure, mmHg, | 158.67(13.29) /89.10(7.75) | 157.52(12.34) /88.31(8.22) | 0.279 /0.308 | 158.01(12.24) /88.33(8.73) | 157.31(12.48) /88.26(8.18) | 0.472 /0.908 |
| Pretreatment NIHSS | | | 0.171 | | | 0.629 |
| 5-7, n (%) | 56(35.9) | 334(28.7) | | 55(27.1) | 253(28.6) | |
| 8-15, n (%) | 73(46.8) | 619(53.2) | | 115(56.7) | 469(53.1) | |
| ≧ 16, n (%) | 27(17.3) | 211(18.1) | | 33(16.3) | 162(18.3) | |
| Time to treatment, h | | | 0.777 | | | 0.771 |
| ≦ 6, n (%) | 9(5.8) | 84(7.2) | | 14(6.9) | 62(7.0) | |
| 7-12, n (%) | 18(11.5) | 144(12.4) | | 22(10.8) | 115(13.0) | |
| 12-24, n (%) | 31(19.9) | 187(16.1) | | 31(15.3) | 144(16.3) | |
| 25-48, n (%) | 51(32.7) | 397(34.1) | | 67(33.0) | 301(34) | |
| 49-72, n (%) | 47(30.1) | 352(30.2) | | 69(34.0) | 262(29.6) | |
| Initial fibrinogen level, g/L, mean (SD) | 3.15(0.95) | 3.19(1.06) | 0.692 | 3.27(1.15) | 3.16(1.01) | 0.165 |
| Initial BI, mean (SD) | 52.08(13.14) | 52.41(13.25) | 0.769 | 51.86(13.60) | 52.96(13.05) | 0.072 |
| Modified Rankin Score | | | 0.205 | | | 0.601 |
| 2 or 3, n (%) | 55(35.2) | 341(29.3) | | 67(33.0) | 257(29.1) | |
| 4 or 5, n (%) | 101(64.7) | 823(70.7) | | 136(67.0) | 595(67.3) | |
| Number of defibrase infusions | | | 0.301 | | | 0.545 |
| 1, n (%) | 17(10.9) | 178(15.3) | | 27(13.3) | 138(15.6) | |
| 2, n (%) | 22(14.1) | 174(14.9) | | 34(16.7) | 127(14.4) | |
| 3, n (%) | 117(75.0) | 812(69.8) | | 142(70.0) | 619(70.0) | |

| | | | | | | |
|---|---|---|---|---|---|---|
| Dose of defibrase | | | 0.108 | | | 0.080 |
| 5 U, n (%) | 4(2.6) | 67(5.8) | | 8(3.9) | 52(5.9) | |
| 10 U, n (%) | 21(13.5) | 131(11.3) | | 17(8.4) | 109(12.3) | |
| 15 U, n (%) | 33(21.2) | 189(16.2) | | 39(19.2) | 136(15.4) | |
| 20 U, n (%) | 57(36.5) | 372(32.0) | | 78(38.4) | 269(30.4) | |
| 25 U, n (%) | 29(18.6) | 292(25.1) | | 44(21.7) | 228(25.8) | |
| 30 U, n (%) | 12(7.7) | 113(9.7) | | 17(8.4) | 90(10.2) | |

Appendix 3 Differences between the patients of loss to follow-up and patients of being followed up at 3 months and 12 months of demographic and baseline clinical charateristics. Abbreviations: SD, standard deviation; Hct=Hematocrit value, PLT=Platelet count, APTT=activated partial thromboplastin time, PT=prothrombin time, TT=thrombin time; TIA = transient ischemic attack; NIHSS, National Institute of Health Stroke Scale

| | Barthel Index (95 or 100) | | mRS (0 or 1) | |
|---|---|---|---|---|
| | 3months | 12 months | 3months | 12 months |
| Initial fibrinogen, n/N, % | | | | |
| 1.5-2.0 g/L | 45/96, 46.9 | 33/66, 50.0 | 26/96, 26.1 | 33/66, 50.0 |
| ≧2.0 g/L | 481/991, 48.5 | 425/708, 60 | 391/991, 39.5* | 424/708, 59.9 |
| Times of defibrase, n/N, % | | | | |
| Once | 75/166, 45.2 | 67/119, 56.3 | 48/166, 41.5 | 67/119, 46.3 |
| Twice | 87/171, 50.99 | 76/120, 63.3 | 71/171, 41.5 | 77/120, 64.1 |
| Three times | 364/750, 48.5 | 315/535,58.9 | 277/750,36.9* | 314/536, 58.6 |
| Dose of defibrase, n/N, % | | | | |
| 5U | 29/81,35.8 | 31/58, 53.4 | 51/81, 63.0 | 40/58, 69.0 |
| 10U | 60/108, 55.6 | 51/81, 63.0 | 20/108, 18.5 | 36/81, 44.4 |
| 15 U | 74/165, 44.8 | 89/122, 73.0 | 42/165, 25.5 | 86/122, 70.5 |
| 20 U | 187/345, 54.2 | 150/232, 64.7 | 147/345, 42.6 | 110/232, 47.4 |
| 25 U | 130/294, 44.2 | 111/219, 50.7 | 141/294, 48.0 | 122/219, 55.7 |
| 30 U | 47/94, 50.0** | 34/64, 53.1** | 17/94, 18.1** | 16/64, 25** |
| Time-to-treat, n/N, % | | | | |
| 0-6h | 37/78, 47.44 | 33/52, 63.46 | 31/78, 39.74 | 23/52, 44.23 |
| 7-12h | 64/134, 47.76 | 59/102, 57.84 | 61/134, 45.52 | 66/102, 64.71 |
| 13-24h | 80/178, 44.94 | 78/128, 60.94 | 68/178, 38.20 | 71/128,55.47 |
| 25-48h | 184/366, 50.27 | 159/264, 60.23 | 138/366, 37.70 | 162/264,61.36 |
| 49-72h | 161/331, 48.64 | 129/229, 56.33 | 119/331, 35.95 | 136/229,59.39 |

Appendix 4. Distribution by percent of patients attained Functional success in different categories; N, the total number of patients followed up at 3months or 12 months; *, $P < 0.05$; **, $P < 0.01$. Abbreviations: mRS=modified Rankin Score
